# *In Utero* Exposure to Environmental Tobacco Smoke Increases Neuroinflammation in Offspring

**DOI:** 10.3389/ftox.2021.802542

**Published:** 2022-01-17

**Authors:** Ana Carolina Cardoso dos Santos Durão, Wesley Nogueira Brandão, Vitor Bruno, Lídia Emmanuela W. Spelta, Stephanie de Oliveira Duro, Nilton Barreto dos Santos, Beatriz Aparecida Passos Bismara Paranhos, Nágela Ghabdan Zanluqui, Maurício Yonamine, Jean Pierre Schatzmann Peron, Carolina Demarchi Munhoz, Tania Marcourakis

**Affiliations:** ^1^ Department of Clinical and Toxicological Analyses, School of Pharmaceutical Sciences, University of São Paulo, São Paulo, Brazil; ^2^ Department of Immunology, Institute of Biomedical Sciences, University of São Paulo, São Paulo, Brazil; ^3^ Department of Pharmacology, Institute of Biomedical Sciences, University of São Paulo, São Paulo, Brazil

**Keywords:** neuroinflammation, pregnancy, experimental autoimmue encephalomyelitis, cell culture, environmental tobacco smoke

## Abstract

The embryonic stage is the most vulnerable period for congenital abnormalities. Due to its prolonged developmental course, the central nervous system (CNS) is susceptible to numerous genetic, epigenetic, and environmental influences. During embryo implantation, the CNS is more vulnerable to external influences such as environmental tobacco smoke (ETS), increasing the risk for delayed fetal growth, sudden infant death syndrome, and immune system abnormalities. This study aimed to evaluate the effects of *in utero* exposure to ETS on neuroinflammation in the offspring of pregnant mice challenged or not with lipopolysaccharide (LPS). After the confirmation of mating by the presence of the vaginal plug until offspring birth, pregnant C57BL/6 mice were exposed to either 3R4F cigarettes smoke (Kentucky University) or compressed air, twice a day (1h each), for 21 days. *Enhanced glial cell* and *mixed cell cultures* were prepared from 3-day-old mouse pups. After cell maturation, both cells were stimulated with LPS or saline. To inhibit microglia activation, minocycline was added to the *mixed cell culture* media 24 h before LPS challenge. To verify the influence of *in utero* exposure to ETS on the development of neuroinflammatory events in adulthood, a different set of 8-week-old animals was submitted to the Autoimmune Experimental Encephalomyelitis (EAE) model. The results indicate that cells from LPS-challenged pups exposed to ETS *in utero* presented high levels of proinflammatory cytokines such as interleukin 6 (IL-6) and tumor necrosis factor-alpha (TNFα) and decreased cell viability. Such a proinflammatory environment could modulate fetal programming by an increase in microglia and astrocytes miRNA155. This scenario may lead to the more severe EAE observed in pups exposed to ETS *in utero*.

## 1 Introduction

Over the course of the 20th century, more than 100 million people died from smoking, and this number is expected to reach one billion in the 21st century (World Health Organization, 2017). Although countries such as Brazil, the United Kingdom, and Australia managed to reduce the numbers of smokers through the implementation of public policies, maternal smoking and passive smoking during pregnancy are still important public health concerns. The effects of prenatal exposure to environmental tobacco smoke (ETS) are similar to those of active maternal smoking (Lin et al., 2017), with more than 12 million babies being exposed to ETS *in utero* each year (Ng et al., 2013).

ETS contains more than 7,000 substances, including nicotine, carbon monoxide, ammonia, benzene, and nitrosamines (Duarte et al., 2006; Ingle et al., 2015) and is composed of both mainstream and sidestream smoke. Differently from mainstream smoke – the one exhaled by the smoker, – sidestream smoke streams from the burning tip of the cigarette, passing through no type of filter (cigarette or lung) and thus being more harmful than mainstream smoke. Moreover, it is formed by incomplete combustion, including greater amounts of toxic and carcinogenic substances per unit of mass (Mello et al., 2001; Organização Pan-Americana da Saúde, 2008; Salmória and Oliveira, 2008).

Oxygen deprivation caused by ETS exposure can modify the proliferation of placental cytotrophoblast cells in critical embryonic developmental stages, besides increasing the risk of postnatal autoimmune diseases such as asthma (Prescott, 2008). In fact, prenatal exposure to ETS can delay fetus growth, increase the risk of sudden infant death syndrome, and promote the development of diseases such as addiction, obesity, and cardiometabolic disorders (Lee and Pausova, 2013).

Regarding the central nervous system (CNS), maternal exposure to ETS is associated with alterations in key neurodevelopmental components, such as the formation of the prefrontal cortex, hippocampus, and striatum, possibly leading to psychological, behavioral, and cognitive changes in adulthood (Lee et al., 2011; Mund et al., 2013). However, few studies assess the effects of ETS exposure on brain inflammation. Khanna et al. (2013) showed that Lewis rats exposed to ETS for 45 days had increased brain proinflammatory genes such as interleukin 6 (IL-6), interleukin 1 beta (IL-1β), and tumor necrosis factor-alpha (TNFα). Although not fully explored, such a phenomenon might result from astrocytes and microglia response.

Glial cells have several different functions in the brain, among which its ability to induce inflammatory responses is the most studied. Resident glial cells are activated with CNS injury, responding by producing cytokines and chemokines for communicating with neurons. With that, peripheral immune cells such as neutrophils, monocytes, and lymphocytes are recruited into the CNS. Despite the possible benefits arising from neuroinflammation, which represents the coordinated response to tissue damage, its persistence can induce a secondary injury, leading to the onset of neurodegenerative diseases (Kraft and Harry, 2011; Prinz and Priller, 2014; Ransohoff, 2016).

Increased levels of cytokines such as IL-1α and TNF-α may produce a neurotoxic effect, causing the apoptotic death of cortical and motor neurons, as well as of mature oligodendrocytes. This constant inflammation state is driven by genetic alterations and environmental factors such as ETS exposure (Goldstein and Kopin, 2007; Medzhitov, 2008) and may be involved in the pathophysiology of neurodegenerative diseases such as Alzheimer’s, Parkinson’s, and Multiple Sclerosis (MS) (Lyman et al., 2014; Rocha et al., 2015; Shemer et al., 2015; Wang et al., 2015; Liddelow et al., 2017).

MS is an autoimmune and neuroinflammatory disease characterized by CNS demyelination. Activation of immune and glial cells plays a crucial role in this disease modulation, contributing to the neurodegeneration observed both in MS and MS animal model – the experimental autoimmune encephalomyelitis (EAE). A recent study suggests that the risk of developing MS is 50% higher for active smokers, 30% higher for passive smokers, and more than twice for children exposed to passive smoking compared to those that don’t smoke. Similarly, mortality risk is two to four times greater among MS patients who are active or former smokers than among those who have never smoked in their lives (Alrouji et al., 2019).

Perturbation of the intrauterine environment has been associated with the development of diseases in adulthood. The concept of fetal programming was first introduced in a study investigating late-life obesity of children born during the Dutch famine, at the end of World War II (Dutch Hunger Winter cohort), highlighting the role of epigenetics (Tobi et al., 2014). Maternal smoking was likewise identified as a long-term risk factor for the development of diabetes and other metabolic disorders (Bergen, 2006). The main epigenetic mechanisms are DNA methylation and histone modifications, and miRNAs have been considered to play a critical role in the control of both processes (Chuang and Jones, 2007).

This study aimed to evaluate whether *in utero* exposure to ETS could modulate glial cells biology and its response to a systemic inflammatory challenge in the offspring. It also investigated whether these alterations are maintained throughout the course of life, as well as their impact on the development of neuroinflammatory/neurodegenerative diseases such as MS, where microglia activation is crucial for disease pathogenesis. Our results show that *in utero* exposure to ETS leads to fetal reprogramming with increased neuroinflammatory response to systemic stimuli, which may have consequences until adulthood, increasing EAE progression.

## 2 Materials and Methods

### 2.1 Animals and Experimental Design

C57BL/6 mice were obtained from the animal facility of the School of Pharmaceutical Sciences of the University of São Paulo and housed at 20–22°C under a 12:12 h light/dark cycle. The animals had access to commercial pellet food for small rodents (Nuvilab^®^ CR-1; Colombo, Brazil) and water *ad libitum*. All procedures were approved by the Ethics Committee of the School of Pharmaceutical Sciences (CEUA/FCF/486) (University of São Paulo). Twenty-eight female mice were allocated to fourteen males, ensuring the housing of two females per each male for 24 h. After the confirmation of mating by the presence of the vaginal plug (G0), fourteen pregnant mice were submitted to two 1-h exposures (1 h at 9 a.m. and 1 h at 4 p.m.) to mainstream and sidestream smoke of 3R4F reference cigarettes (College of Agriculture, University of Kentucky) until delivery (G21; average = 5 pups per female). Exposure was performed in a chamber measuring 564 mm width × 385 mm depth × 371 mm height, in which carbon monoxide (CO) level was measured every 10 min using a gas detector (ToxiPro, Biosystems, United States). Fourteen control pregnant mice were placed in a similar chamber and exposed to compressed air at 2 ml/min (pressure: 4 bar) (Lobo-Torres et al., 2012; Torres et al., 2019a; Torres et al., 2020). A different group of females (*n* = 6) was also exposed to compressed air and ETS, having blood samples collected from their inferior vena cava immediately after the last exposure to quantify biological exposure markers. Carboxyhemoglobin (COHb) was analyzed in whole blood using the spectrophotometric method (Beutler and West, 1984), and plasma cotinine and 3-hydroxycotinine quantification was performed by liquid chromatography coupled to tandem mass spectrometry (LC-MS/MS) (Torres et al., 2015a; Torres et al., 2019a).

On the third day of life (P3), the animals from both sexes were decapitated, and two types of cultures were prepared from the whole brain of neonates: an enhanced glial cells culture (*enhanced glial cell culture*) and a mixed glia/neuron cell culture (*mixed cell culture).* Each neonate was considered an individual experimental number. Upon maturation, cultures were stimulated with 100 ng/ml of lipopolysaccharide (LPS) from *Escherichia coli* (0111: B4, Sigma) to stimulate immune system. Experiments were performed after 24 h. The *mixed cell culture* was incubated with minocycline (10 μM) 24 h before LPS stimulation to inhibit microglia activation.

**FIGURE 1:**
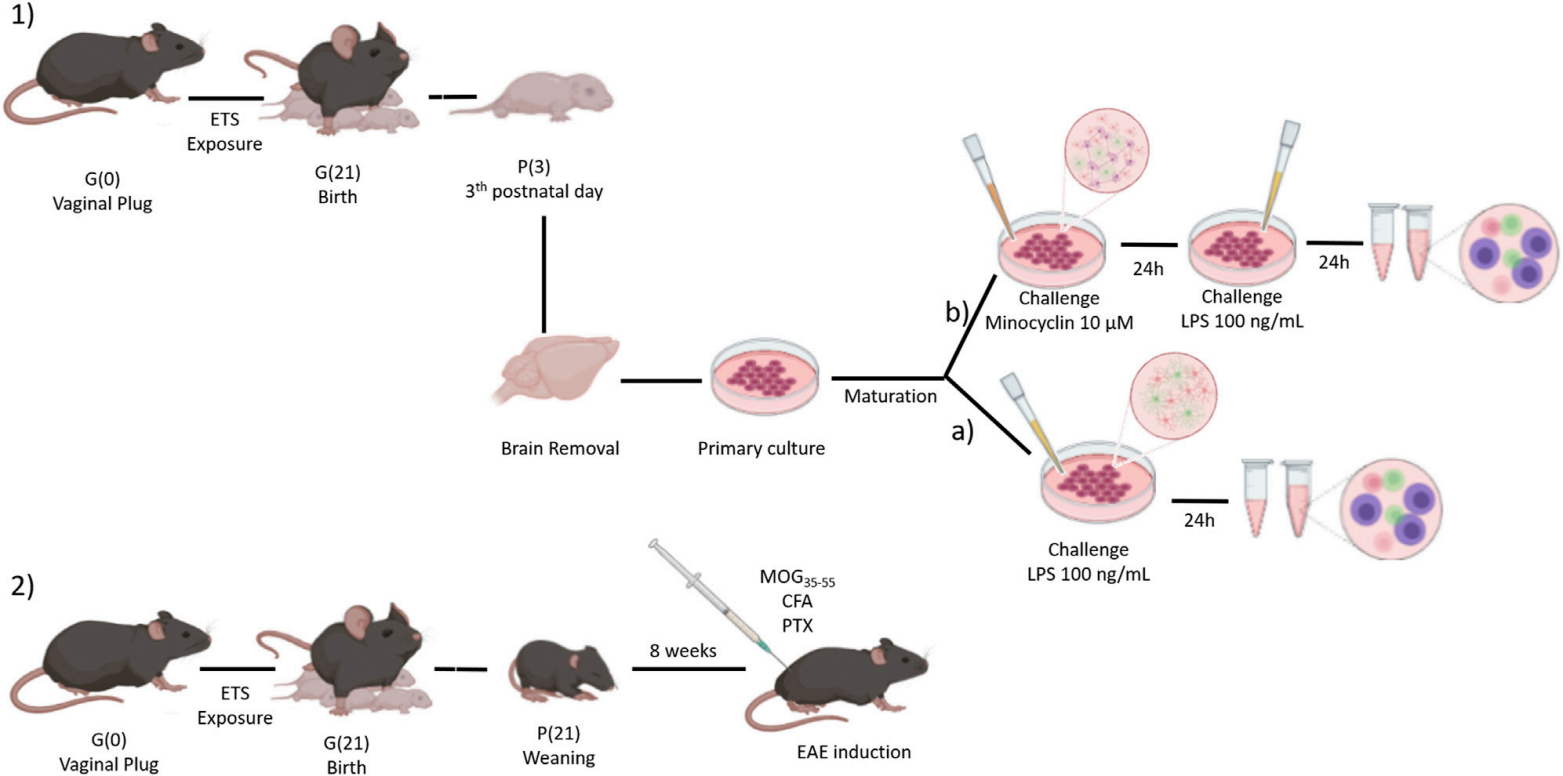
Female mice were exposed to environmental tobacco smoke from confirmation of mating by vaginal plug until offspring birth. 1) On the third day of postnatal life (P3), the pup’s central nervous system was removed and used to prepare the cell cultures. **(A)** *enhanced glial cell culture* was maturated for 21 days and then stimulated with LPS 100 ng/ml for 24 h before experimental analyses. **(B)** *mixed cell culture* was maturated for 7 days and then stimulated with minocycline (10 µM, 24 h) and challenged with LPS 100 ng/ml for 24 h before experimental analyses. 2) After weaning, animals were kept in their cage for 8 weeks, when they were submitted to an experimental autoimmune encephalomyelitis (EAE) protocol. Figure made at BioRender.com.

To verify the influence of *in utero* exposure to ETS in the development of neuroinflammatory events during adulthood, a set of animals was kept at their home cages for 8 weeks after weaning and then submitted to the Experimental Autoimmune Encephalomyelitis (EAE) protocol. The animals were euthanized by decapitation after anesthesia with ketamine and xylazine. The CNS and spinal cord were immediately removed and stored at −80°C. Figure 1 presents the experimental protocol, conducted with the following experimental groups*:*

✓ Control Group (CO) – whole-brain cell cultures of the offspring of females exposed *in utero* to compressed air and challenged with culture medium.
✓ Smoker Group (ETS) – whole-brain cell cultures of the offspring of females exposed *in utero* to environmental tobacco smoke and challenged with culture medium.
✓ Group LPS (LPS) – whole-brain cell cultures of the offspring of females exposed *in utero* to compressed air and challenged with LPS.
✓ Smoker Group + LPS (EPS) – whole-brain cell cultures of the offspring of females exposed *in utero* to environmental tobacco smoke and challenged with LPS.

### 2.2 Cell Cultures

Cell cultures were prepared according to Saura et al. (2003). After removing the brains of 3-day-old animals, the meninges were extracted with the aid of a magnifying glass. Brain tissue was incubated with trypsin (0.25%) for 10 min at 37°C, supplemented with 10% fetal bovine serum (FBS), and processed with pipettes of different size for mechanical digestion. After 2 min, and the supernatant was collected and filtered through a 70 μM membrane. The suspension was then centrifuged at 450 × g for 5 min at room temperature and the pellet was resuspended in DMEN-F12 containing 10% FBS, 1% L-glutamine (200 mM), 1% nonessential amino acids, 1% pyruvate, and 1% antibiotic (penicillin and streptomycin). Cells were grown on plates of different diameters, according with the experiment to be performed.

For the *mixed cell culture*, plates were previously treated with Poly-L-lysine for 3 h in a 37°C oven and later conditioned with the medium (DMEN-F12 containing 10% FBS, 1% L-glutamine (200 mM), 1% nonessential amino acids, 1% pyruvate, and 1% antibiotic (penicillin and streptomycin)) for 30 min. Then, cells were plated and incubated for 7 days at 37°C with 5% CO_2_. The *enhanced glial cell culture* was plated and incubated at 37°C with 5% CO_2_ for approximately 21 days until confluence.

### 2.3 Water and Food Intake and Weight Evaluation

Pregnant mice were weighed from the first day of exposure (day 0) until the 20th gestational day, always before morning exposure. Water and food intake were evaluated every 5 days, until the 20th day. The average intake throughout the experimental period was determined per animal per box. Neonates were individually weighed on the 3^rd^ day of life before euthanasia.

### 2.4 Cell Viability, Cytokine Concentration, and Cell Markers

To prevent non-specific antibody binding, 1 × 10^6^ cells from *enhanced glial cell culture* (*n* = 6) were resuspended in 80 μl blocking solution (Fc blocking) and incubated for 20 min at 4°C. Then, cells were centrifuged at 450 × g and incubated with the CD80 – PE, CD86 – PERCP, and CD11b – APC antibodies for 20 min at 4°C. After being washed twice with 200 μl PBS and centrifuged at 450 × g at 4 °C for 5 min, samples were resuspended in 100 μl of Fixperm^®^, incubated for 20 min at 4°C, washed with 100 μl Permwash^®^, and centrifuged at 450 × g at 4°C for 5 min. Cells were then incubated with the desired antibody (GFAP - FITC), washed, centrifuged at 450 × g at 4°C for 5 min, and resuspended in 200 μl 1% paraformaldehyde.

In the *mixed cell culture* (*n* = 5), apoptosis was verified by Annexin and propidium iodide (PI) assay. Cells incubated with trypsin (0.25%) were collected, washed, and resuspended in binding buffer (10 mM Hepes - pH 7.4, 150 mM NaCl, 5 mM KCl, 1 mM MgCl_2_, and 1.8 mM CaCl_2_) containing annexin V-FITC and PI at 1:20 dilution (in a final volume of 20 μl). After being incubated at room temperature for 20 min in the dark, 80 μl of the binding buffer was added to the samples.

IL-1β, IL-6, IL-12, IFN-γ, IL-10, MP2, and TNF-α cytokines in cell supernatants were quantified in both *enhanced glial cell culture* (*n* = 6) and *mixed cell culture* (*n* = 5) using the cytometry beads array (CBA) according to the manufacturer’s instructions (BD^®^). Samples were collected and analyzed on a FACS Accuri C6^®^ flow cytometer (Becton & Dickinson, Mountain View, CA).

### 2.5 Immunofluorescence Characterization of Cultured Cells

After the incubation period, culture medium was removed and cells were washed three times with PBS, fixed with methanol 100% for 20 min at room temperature, and washed with PBS again. Then, cells were incubated in 40 mM glycine solution in PBS for 5 min and washed. Non-specific sites were blocked with 0.05% Triton, 1% bovine serum albumin (BSA) in PBS for 2 h at room temperature. After washing, cells were incubated with specific primary antibodies (GFAP anti-mouse-Alexa fluorine 594 (1:500); MAP2 anti-chicken-Alexa fluorine 555 (1:250); and microglia tomato-lectin-FITC (1:500) diluted in blocking solution) overnight at 4°C on a dark chamber. Then, cells were washed five times with PBS for 5 min and incubated for 2 h at room temperature on a dark chamber with a specific secondary antibody. After washing the cells five times with PBS, 150 μl DAPI (1:100.000) was added to samples for nuclear visualization. Slides were analyzed using the ZOE™ Fluorescent Cell Imager microscope.

### 2.6 Nitric Oxide Quantification

The *mixed cell culture* supernatant (*n* = 5) and the Griess reagent (0.1% N-1 Naphthyl-ethylenediamine, 1% sulfanilamide, 2.5% H_3_PO_4_) were added to a 96-well plate in equal proportions (1:1). Samples were incubated for 10 min at room temperature, and absorbance was measured at 550 nm. The analyses were compared with a NaNO_2_ (0–100 μM) standard curve.

### 2.7 Real-Time PCR

For quantitative real-time polymerase chain reaction (qRT PCR), total mRNA was isolated from *enhanced glial cell culture* (*n* = 3) with Triazole. Using Invitrogen Superscript III, mRNA (1 µg/ml) was converted into cDNA and diluted in DEPC-treated water in the proportion of 1:5 using Applied Biosystems Taqman probes with Taqman Universal Master Mix II, according to the manufacturer’s instructions. The qPCR was performed on the Applied Biosystems QuantStudio™ 3 for *IL-6* (Mm00446190_m1)*, iNOS* (Mm00440502_m1)*, TLR4* (Mm00445273_m1) and *Chuk* (IKKα - Mm00432543_g1), considering *β-actin* (Mm02619580_g1) as a housekeeping gene. The relative fold expression of mRNA was determined using the ^∆∆^Ct method. All readings were made in duplicate.

### 2.8 miRNA Quantification

RNA from the *enhanced glial cell culture* (*n* = 3) was extracted as described above. The cDNA synthesis was performed on 130 ng of total RNA by means of a polyadenylation reaction followed by a reverse transcription reaction. Gene expression was quantified by real-time PCR using the Stratagene Mx3005P equipment from Agilent Technologies (Santa Clara, California, United States), SYBR Green as fluorescent marker (Luna^®^ Universal qPCR Master Mix #M3003L), and universal antisense primer (each miRNA specific primer was used as sense). The expression of miR-146a, miR-155 and miR-223 were quantified using the comparative cycle threshold (Ct) method, and results were normalized with the constitutive gene RNU43. All readings were made in duplicate.

### 2.9 Experimental Autoimmune Encephalomyelitis

To induce EAE, 8-week-old male mice exposed (*n* = 9) or not (*n* = 11) to ETS *in utero* were subcutaneously injected into the hind flank with 150 μg MOG_35–55_ peptide (MEVGWYRSPFSRVVHLYRNGK, synthesized by Proteimax Biotechnology - São Paulo - SP - Brazil), emulsified in complete Freund’s adjuvant (v/v) containing 5 mg/ml of BCG (*Bacillus Calmette-Guérin*). On the day of inoculation and 2 days after, mice received intraperitoneal injections of 200 ng pertussis toxin. Mice were monitored daily for symptoms of EAE, classified according to an arbitrary scale ranging from 0 to 5, whereby 0 indicates no symptoms; 1 indicates flaccid tail; 2 hindlimb weakness or abnormal gait; 3 total hindlimb paralysis; 4 total hindlimb paralysis with forelimb weakness or paralysis; and 5 moribund or deceased mice.

After euthanasia with ketamine (500 mg/kg i.p.) and xylazine (50 mg/kg i.p), spinal cord samples were removed from animals and fixed in buffered formalin. Inflammation was evaluated by analyzing inflammatory infiltrates in Hematoxylin & Eosin (H&E) staining sections and demyelination by Luxol Fast Blue (LFB) staining. The semiquantitative evaluation was adapted from Soellner et al. (2013).

### 2.10 Statistical Analysis

*In vivo* animals and the *enhanced glial cell culture* were analyzed by means of a two-way analysis of variance (ANOVA) with the subject factor’s “exposure” (ETS or compressed air *in utero*) and “challenge.” Microglia contribution in *mixed cell culture* results was evaluated using a tree-way ANOVA, considering the factors “exposure,” “challenge,” and “minocycline” (present or absent). Multiple comparisons were performed by the Tukey’s post-hoc test, and *p* < 0.05 was considered as statistically significant. Values are expressed as mean ± standard error of the mean (SEM).

## 3 Results

During exposure, CO levels in the chamber were equal to 470.2 ± 90.93 ppm and exposure biomarkers measurements were as follows: COHb: 21.62 ± 1.80%; plasma cotinine: 139.94 ± 13.02 ng/ml; and plasma 3-hydroxycotine: 113.65 ± 16.78 ng/ml. Supplementary Figure S1 shows the food intake of pregnant mice, as well as their weight during pregnancy and the weight of the offspring. When compared with mice exposed to compressed air, pregnant females exposed to tobacco smoke ingested 24% less food and gained less weight during gestation. Moreover, the offspring exposed *in utero* to ETS showed less weight gain than the control group.

### 3.1 Exposure to Environmental Tobacco Smoke Increases Glial Cell Response to Inflammatory Stimuli

As shown in Supplementary Figure S2, the enhanced glial cell culture was composed of 70% astrocytes and 30% microglia cells.

**FIGURE 2:**
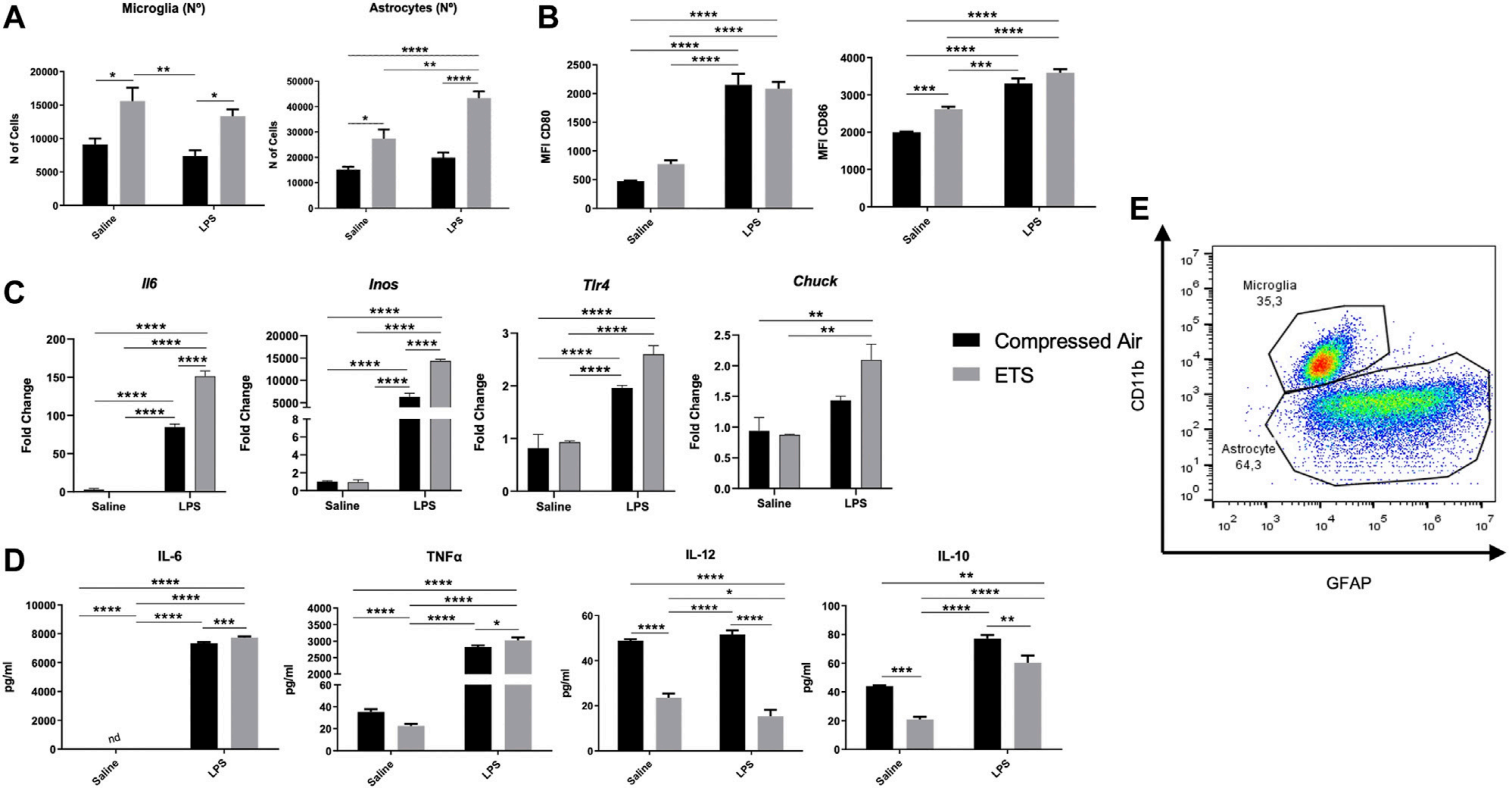
*Enhanced glia cell culture* from C57Bl/6 mice offspring exposed or not to environmental tobacco smoke *in utero*. After maturation, cells were challenged or not with LPS. **(A)** Flow cytometry analysis of microglia (CD11b) and astrocytes (GFAP) in *enhanced glia cell culture,* graphs for the total number of cells; **(B)** mean fluorescence intensity (MFI) for CD80 and CD86; **(C)** Gene expression analysis by PCR; **(D)** Cytokine’s quantification in culture supernatant; **(E)** Gate representative of cell frequency in *enhanced glia cell culture.* Statistical analysis: Two-way ANOVA and Tukey’s post-hoc. Data are expressed as mean ± SEM. *p < 0.05, **p < 0.01, ***p < 0.001, ****p < 0.0001. The black bar represents exposure to compressed air and gray bar represents exposure to ETS.

The number of astrocytes within the enhanced glial cell culture was investigated by two-way ANOVA (Supplementary Table S1). Tukey’s post-hoc analysis showed a higher number of astrocytes in the brain cells of mice exposed *in utero* to ETS (*p* < 0.05) when compared with control. The group exposed to ETS and challenged with LPS had increased astrocytes levels compared to CO (*p* < 0.0001), ETS (*p* < 0.01), and LPS (*p* < 0.0001) (Figure 2A). Regarding microglia, the Tukey’s post-hoc analysis revealed an increase in microglia cells of the group exposed *in utero* to ETS, regardless of challenge with (*p* < 0.05) or without LPS (*p* < 0.001) (Figure 2A).

Microglia activation by LPS was verified by measuring the CD80 and CD86 costimulatory molecules. For CD80, Tukey’s post-hoc analysis showed higher levels for groups challenged with LPS when compared with the CO (*p* < 0.0001) and ETS (*p* < 0.0001), despite ETS exposure. As for CD86, post-hoc test indicates an increase in this molecule in LPS-challenged groups when compared with CO (*p* < 0.0001) and ETS (*p* < 0.0001). CD86 levels where higher in the group only exposed to ETS in relation to the control (*p* < 0.01) (Figure 2B).

We also verified glial activation by LPS through enhanced gene expression and cytokines release. Interaction between “exposure” × “challenge” was considered significant for Il-6 and iNOS (Supplementary Table S1). When compared with ETS and CO groups (*p* < 0.0001), Il-6, *Inos*, and Tlr4 presented increased expression in groups challenged with LPS regardless of exposure to ETS, as verified by Tukey’s post-hoc test. Furthermore, groups challenged with LPS and exposed to ETS showed increased levels of Il-6 and *Inos* when compared to the group only challenged with LPS (*p* < 0.0001). The higher expression of IKKα (Chuck), the protein responsible for the phosphorylation of IκB and NF-κB dimer release to the nucleus, allow us to infer a higher activity of NFκB in the EPS group (CO and ETS *p* < 0.05 for both) (Figure 2C).

Complementing the results obtained by the qPCR, Figure 2D shows the results of the two-way ANOVA (Supplementary Table S1) for cytokines release. Tukey’s post-hoc analysis revealed an increase in IL-6 and TNF*α* release in ETS and EPS groups in relation to their respective controls (*p* < 0.0001; *p* < 0.001, respectively), thus suggesting that cells exposed to ETS before LPS challenge have a higher inflammatory capacity. ETS-exposed groups challenged or not with LPS presented a decreased of cytokine IL-12 – which plays an essential role in the polarization of TH1 CD4^+^ T lymphocytes – in relation to control (*p* < 0.0001; *p* < 0.0001 respectively) and LPS group (*p* < 0.0001; *p* < 0.0001, respectively). By comparing both groups exposed to ETS, we observed lower levels of IL-12 release in groups challenged with LPS (*p* < 0.05).

As expected, we verified an increased production of IL-10, an anti-inflammatory cytokine, in the LPS group when compared with the control group (*p* < 0.0001). In turn, the cytokine production decreased in ETS-exposed groups (ETS and EPS) when compared with control (*p* < 0.001, *p* < 0.01) and LPS (*p* < 0.0001, *p* < 0.01). IL-10 release was reduced in the EPS group in relation to LPS group (*p* < 0.0001). These findings suggest that ETS exposure can increase the number of glial cells and their activation, as well as the production and release of proinflammatory cytokines after LPS challenge.

### 3.2 Increased Inflammatory Response by Glial Cells Caused by Environmental Tobacco Smoke Exposure Increases Cell Death

After verifying an increased inflammatory response by glial cells in ETS-exposed groups challenged with LPS, we investigated whether this inflammation process could change cellular viability in a mixed cell culture composed of 3% microglia cells, 39% astrocytes, and 58% neurons (Supplementary Figure S3). To investigate the role of immune cells in this phenomenon, the culture was treated with minocycline 24 h before LPS stimulation, inhibiting microglia activation. Thus, experiments were conducted in the presence or absence of microglia.

**FIGURE 3:**
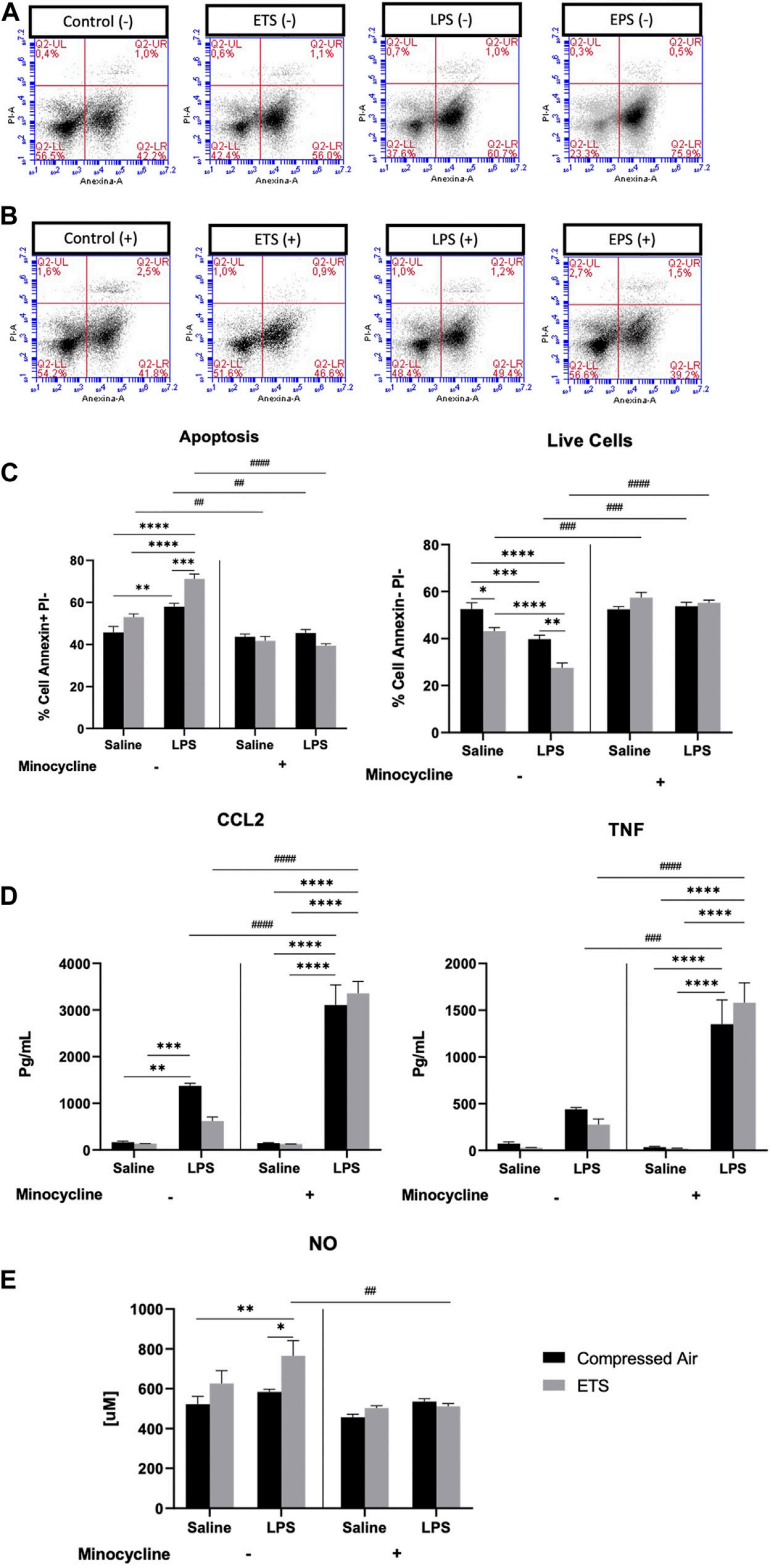
*Mixed cell culture* (mixed neuron-glia culture) from animals exposed or not to environmental tobacco smoke. After maturation, cells were treated with minocycline 24 h before LPS challenge, forming the groups. **(A)** Gate representative of Annexin and PI marking without minocycline. **(B)** Gate representative of Annexin and PI marking with minocycline. **(C)** Annexin and PI analysis, graph for percentage of live cells and apoptosis. **(D)** Cytokine measurement in the supernatant. **(E)** NO determination. Statistical analysis: Three-way ANOVA and Tukey’s post-hoc. Data are expressed as mean ± SEM. *p < 0.05, **p < 0.01, ***p < 0.001, ****p < 0.0001, # shows difference between negative and positive minocycline. The black bar represents exposure to compressed air and gray bar represents exposure to ETS.

Apoptosis and the number of live cells were evaluated by a three-way ANOVA (Supplementary Table S2). The results indicate a significant effect of the interactions between minocycline and “challenge”. However, we found no interaction between all the factors (“challenge”, “exposure” and “minocycline”). Tukey’s post-hoc test showed that all groups presented a decrease in cell viability (*p* < 0.0001) in the presence of a functional microglia when compared with control (Figure 3C). These results are in line with the increased percentage of apoptotic cells (*p* < 0.0001) in LPS (*p* < 0.01) and EPS (*p* < 0.0001) in relation to control. Moreover, the EPS group showed a decrease in cell viability when compared with ETS groups (*p* < 0.0001). Corroborating this finding, the number of apoptotic cells was higher in the EPS group than in the ETS group (*p* < 0.0001). These results suggest that previous exposure to ETS reduce cell viability and increase apoptosis in relation to LPS challenge alone.

To understand the mechanism controlling microglia apoptosis, we measured the production of TNFα, CCL2 (Figure 3D), and NO (Figure 3E) with and without the presence of minocycline. Tukey’s post-hoc test demonstrated an increase in CCL2 (*p* < 0.0001) and TNF-α release (*p* < 0.001) in the LPS-challenged group with minocycline when compared with its respective experimental group without minocycline, showing the effect of astrocyte in releasing these cytokines in the absence of microglia. The same result was observed regarding the EPS group for CCL2 (*p* < 0.0001) and TNF-α (*p* < 0.0001). In the presence of microglia, CCL2 release increases in the LPS group when compared with CO (*p* < 0.01) and ETS groups (*p* < 0.001).

When assessing NO production, the Tukey’s post-hoc test showed an increased release in the EPS group in relation to control (*p* < 0.01) and LPS groups (*p* < 0.05), suggesting that the combination of ETS and LPS increases the activity of microglia cells (Figure 3E). NO release decreased when microglia cells were inhibited by minocycline (*p* < 0.01), corroborating the findings for apoptosis. Thus, we may infer that apoptosis is caused by microglia cells ability to release NO against stimuli.

### 3.3 Increased Glial Response Is Correlated With Changes in miRNA

**FIGURE 4:**
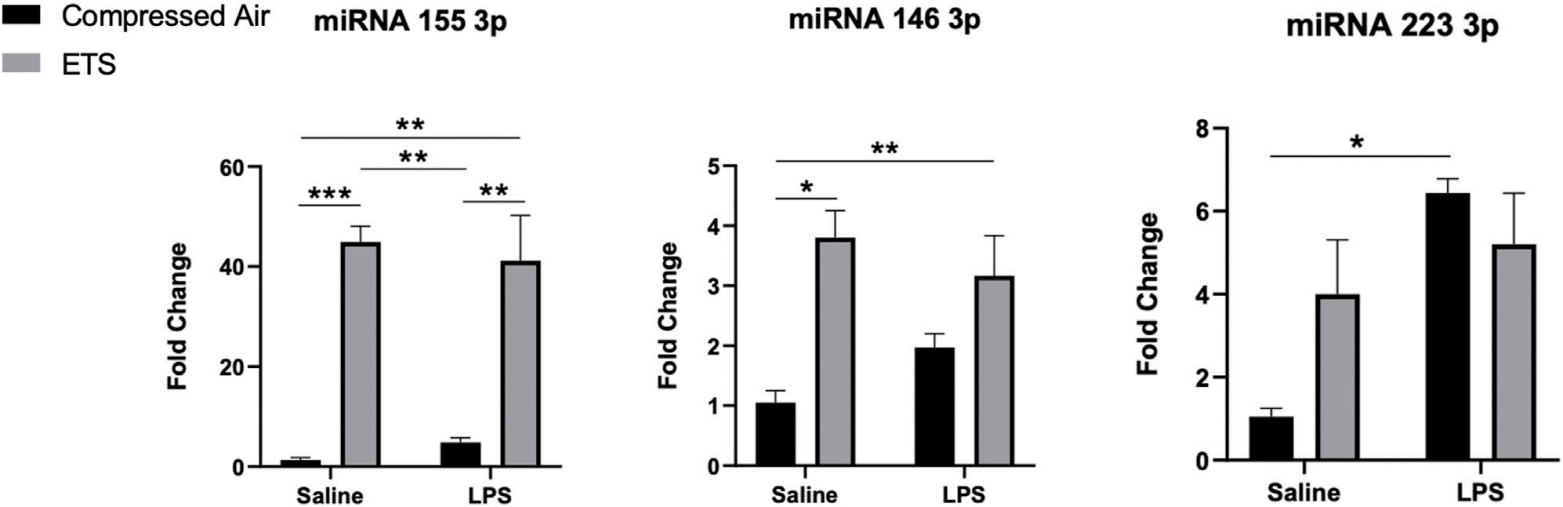
Evaluation of miRNA in *enhanced glial cell culture* from C57Bl/6 mice offspring exposed or not to environmental tobacco smoke *in utero*. After maturation, cells were challenged or not with LPS. Statistical analysis: Two-way ANOVA and Tukey’s post-hoc. Data are expressed as mean ± SEM. *p < 0.05, **p < 0.01, ***p < 0.001, ****p < 0.0001. The black bar represents exposure to compressed air and gray bar represents exposure to ETS.

The miRNAs quantification was performed on *enhanced glial cell culture* cells. Supplementary Table S1 shows the results of the two-way ANOVA. We verified an increase of miR-146 transcription in the ETS-exposed group when compared with the control group (*p* < 0.05), and of miR-155 compared to both control (*p* < 0.05) and LPS groups (*p* < 0.05) (Figure 4). In turn, the ETS-exposed group challenged with LPS showed an increase in miRNA155 transcription in relation to control (*p* < 0.05) and LPS groups (*p* < 0.05), thus indicating the effect only of ETS in these miRNAs. For miR-223, we observed an increased transcription only in the LPS group in relation to control (*p* < 0.05), with no effect of ETS exposure.

### 3.4 *In Utero* Exposure to Environmental Tobacco Smoke Increases Disease Severity in Adult Mice Submitted to Experimental Autoimmune Encephalomyelitis Protocol

To verify the influence of *in utero* exposure to ETS in the development of neuroinflammatory events during adulthood, animals were submitted to the EAE protocol – a murine model of MS.

**FIGURE 5:**
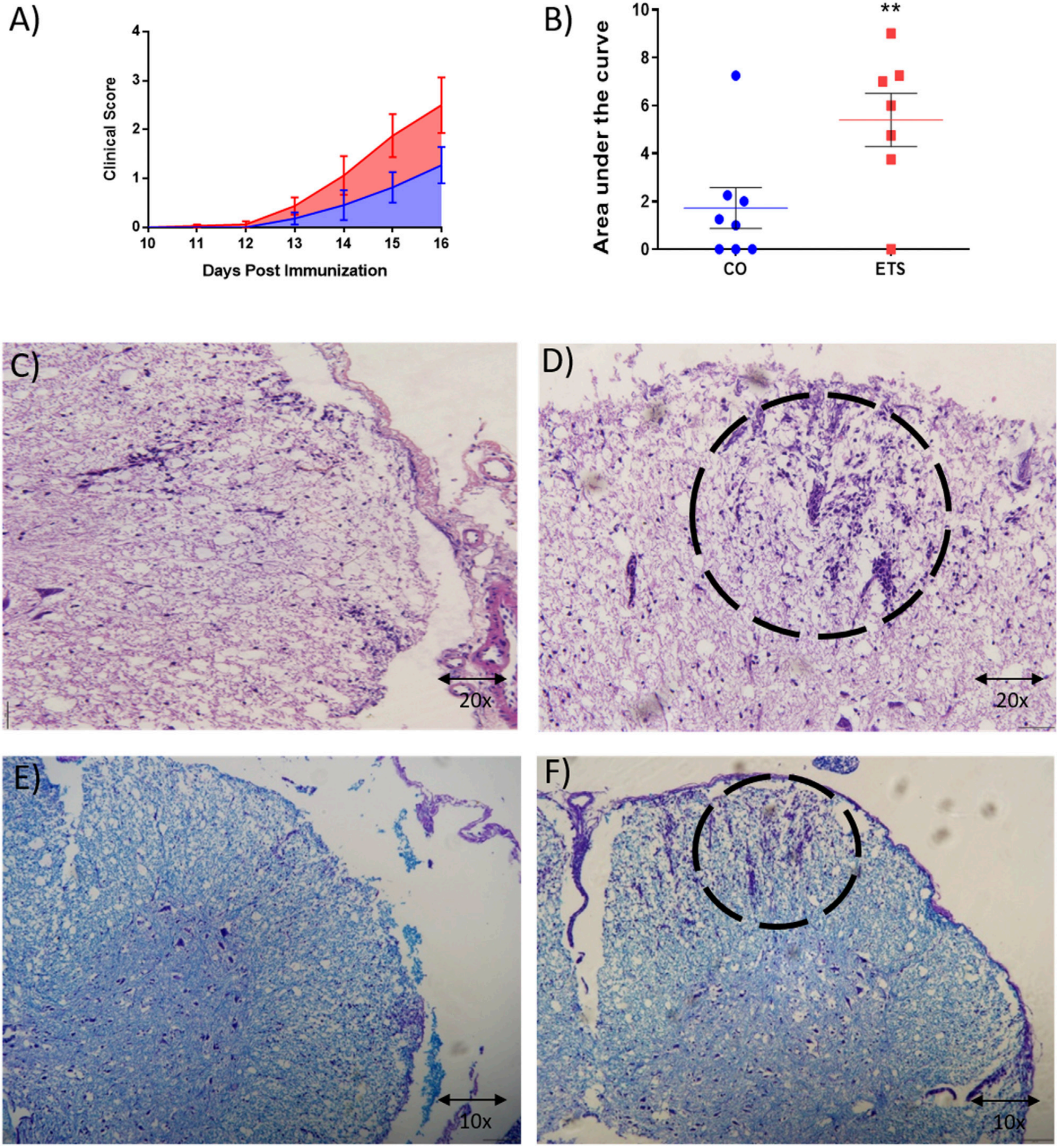
EAE clinical score in animals exposed or not to environmental tobacco smoke **(A)** Curve up to the peak (15th day); **(B)** Area under the curve. Statistical analysis: Student’s t-test *p < 0.05, **p < 0.01, ***p < 0.0001 *n* = 12; **(C–F)** Hematoxylin & Eosin or Luxol Fast Blue staining *n* = 3 **(C,E)** represents control groups and **(D,F)** exposed groups. Marrow cut in the distal third. Data are expressed as mean ± SEM.

As shown in Figure 5A, EAE clinical scores were higher among mice exposed *in utero* to ETS than in the control group, presenting with more exacerbated motor difficulties such as paralysis of both hind paws. The qualitative analysis of cellular infiltrate in the spinal cord of these animals (Figures 5C–F) indicates increased inflammation, possibly associated with EAE clinical signs. Figure 5B shows the area under the curve of clinical scores. These data suggest that the susceptibility of glial cells to inflammatory stimuli caused by *in utero* exposure remains in adulthood and may favor the development of neuroinflammatory diseases such as MS.

## 4 Discussion

Despite all efforts exerted by public health systems worldwide, *in utero* exposure to ETS remains a major concern. Considering that, this study investigated the effects of *in utero* exposure to ETS using *enhanced glial cell culture* and *mixed cell culture* to understand how these cell types react in the presence or absence of an inflammatory stimulus. We verified increased levels of proinflammatory cytokines such as IL-6 and TNFα, as well as decreased viability in the cells of ETS-exposed offspring challenged with LPS, indicating an increase in neuroinflammation. Moreover, pups exposed to ETS presented with more severe EAE, suggesting a fetal reprograming possibly related to the increase in miR-155 observed on the *enhanced glial cell culture* exposed to ETS.

A previous study conducted by our research group showed that ETS exposure induces oxidative stress in several brain regions in adult mice (Lobo Torres et al., 2012). Moreover, early postnatal exposure to ETS impairs myelination, learning, and memory, besides decreasing brain-derived neurotrophic factor (BDNF) and synaptic proteins levels (Toledo-Rodriguez et al., 2010; Torres et al., 2015a,b). In a study conducted by Torres et al. (2020), the authors found that ETS exposure during the early postnatal period decreased ^18^fluorine-fluorodeoxyglucose (^18^F-FDG) uptake, a marker of brain metabolic activity, in several brain areas of males and females from late infancy to early adulthood.

The exposure biomarkers found in this study are consistent with those reported in previous studies of our research group (Lobo Torres et al., 2012; Torres et al., 2015a, 2015b; Torres et al., 2019a; Torres et al., 2019b; Torres et al., 2020), as well as with those of other studies (Mello et al., 2001). Due to the rapid metabolization of nicotine, cotinine and 3-hydroxycotinine were used as biological biomarkers of ETS exposure (Man et al., 2006). According to Gatzke-Kopp et al., 2019, serum cotinine levels can be used to discriminate passive and active smoking, whereby passive smokers can have serum cotinine levels between 0.05 and 10 ng/ml and active smokers >10 ng/ml. However, this classification was based on children and not rodents – which explains why the cotinine levels observed in our study could be considered as the produced by active smokers. Moreover, the study did not mention how long after cigarette exposure the blood was collected, which is an important issue to be considered. In our study, blood samples for biological marker quantification were collected immediately after ETS exposure, so that cotinine levels reflected the peak, as plasma nicotine half-life in rodents is 0.9–1.1 h.

The association between exposure to tobacco smoke, food intake, and weight gain is already well established in the literature and aligned with our results. Chen et al. (2006) showed that exposure to ETS reduced food consumption by about 31% on the first day of exposure. Such an effect may be explained by the nicotine-induced Y neuropeptide decrease in the paraventricular nucleus. We also found a significant difference in the weight of offspring born to females exposed to ETS compared to those of the control group, corroborating the results reported by Obot et al. (2004), Salmória and Oliveira (2008), and Lobo Torres et al., 2012. Low birthweight may have significant negative effects on infants, being associated with higher morbidity and mortality rates.

All CNS cells are involved in neuroinflammation, and microglia activation is the first indication of an insult. Astrocytes and neurons release chemokines to recruit microglia, which is activated from its M2 anti-inflammatory profile to M1 pro-inflammatory profile. Microglia activation can be initiated by LPS binding to the toll-like receptor 4 (TLR4), which increases the expression of MHCII, CD80, and CD86 – costimulatory molecules responsible for APCs communication with lymphocytes (Ewing et al., 2013). When the lesioned site is reached, microglia express and/or activate molecules that enable antigen recognition and presentation on their surface. Once activated, microglia proliferate, migrate, and release mediators such as cytokines and chemokines (e.g. IL-6 and TNFα), besides increasing the production of reactive oxygen/nitrogen species (ROS/RNS) to resolve the inflammatory process (von Bernhardi et al., 2015). Although this study focused on evaluating the effects of *in utero* exposure to ETS in CNS cells *in vitro*, we detected many features present in a neuroinflammatory response, such as the increased expression of the microglia activation marker B7.2 (CD86), as well as the increase in apoptosis of IL-6, TNFα, and NO, and the decrease of live cells in the ETS-exposed group challenged with LPS. These findings suggest that *in utero* exposure to ETS increases microglia proinflammatory response in the offspring.

Transient cell activation in the CNS is beneficial to the organism. For example, IL1 receptor activation is essential for the repopulation of depleted microglia in the CNS (Bruttger et al., 2015)*.* However, the responsive cells persistent activation in the CNS can be harmful, often leading to neuronal death, as observed in neurodegenerative diseases such as Alzheimer’s and MS (Pekny et al., 2016).

We verified that microglia are responsible for the higher frequency of apoptotic cells, mainly by NO, which increases neuroinflammation. NO production and release by microglia iNOS activation can cause neuronal death by preventing cellular respiration, increasing glutamate release, and consequently leading to NMDA receptor-mediated excitotoxicity. Because of the large amounts of iNOS required to cause excitotoxicity, NO may bind to the superoxide anion released by NADPH oxidase from microglia cells, forming peroxynitrite anion – which contributes to apoptosis (Lyman et al., 2014).

The dosages of cytokines with minocycline suggest that astrocytes play a role in this process. Studies from the last 20 years indicate that, despite their essential role in maintaining tissue homeostasis, astrocytes can respond to CNS insults by producing inflammatory cytokines and activating the NFκB pathway – events related to a worse prognosis of neuroinflammatory diseases (Sofroniew, 2015). Mishra et al. (2012) have shown that progressive IL-1β release can reduce the number of synaptic connections between hippocampal neurons in rat culture by increasing glutamate release.

After verifying that *in utero* exposure to ETS elicits neuroinflammation in *in vitro* models, the next step was to investigate whether it could worsen a neurodegenerative disease, to which end we chose the EAE model for the human inflammatory demyelinating disease: multiple sclerosis. EAE mimics many pathological features of MS, such as inflammation, demyelination, axonal loss, and gliosis (Constantinescu et al., 2011). MS is a neuroinflammatory disease characterized by increased inflammation in the CNS, with oligodendrocyte death, progressive demyelination of the white matter, and formation of plaques characteristic of cellular infiltrate (Lyman et al., 2014). Neurological symptoms can vary in severity and typically affect sensory (tingling, numbness, dizziness, and visual disturbances) and motor systems (weakness, difficulty walking, tremors, lack of coordination, and difficulty with excretory controls) (Bronner et al., 2010).

Tobacco smoke is a well-established risk factor not only for MS onset, but also for developing progressive forms of the disease (Waubant et al., 2019). Although the overall frequency of MS is in females (3:1), males are most likely to develop a rapidly disability progression disease (Zhou et al., 2020) with the onset on ages from 25 to 64 years old (Tutuncu et al., 2013). According to Centers for Disease Control and Prevention (2021) the tobacco consumption is higher in males than females (15.3–12.7%), with higher incidences in the ages between 25 and 64 years. These factors together corroborate our choice to work with male animals.

We observed an increase in disease severity upon *in utero* exposure to ETS, indicating that it affected clinical response and cellular activity – which corroborates the different pattern of cellular infiltration and demyelination described by Peron et al. (2012). The worse outcome verified for ETS-exposed offspring suggests a fetal programming during intrauterine life. Epigenetic modifications during gestation, which are necessary for fetal programming, influence organ and tissues development, leading to increased risk for neurodegenerative diseases (Barlow et al., 2007; Faa et al., 2016). During intrauterine life, external influences such as exposure to ETS may modify the epigenetic patterns of fetuses (Ivorra et al., 2015). Piras et al. (2014) suggest that epigenetics may modulate neuronal development, resulting in interindividual variability in the number of neurons and glial cells at birth. miRNAs affect the protein levels of the target without modifying gene sequences, thus being considered as epigenetic modulators. The correlation between miRNAs and CNS is relevant in all periods of life, influencing cell differentiation, proliferation, and synaptogenesis, besides functioning as a biomarker for neurodegenerative diseases. Being the main targets of miRNAs in neuroinflammation (Gaudet et al., 2018), microglia cells and astrocytes play a key role in modulating immune response and thus can be considered as pro- (miRNA-155, miRNA-27b, miRNA-326) or anti-inflammatory (miRNA-223, miRNA-146a) (Chen et al., 2019; Gharibi et al., 2019; Ponnusamy and Yip, 2019).

The *enhanced glial cell culture* of both groups exposed *in utero* to ETS, regardless of challenge with LPS, presented an increase in miR-155 – a miRNA responsible for regulating the function of B lymphocytes and producing cytokines. The levels of miR-155 in astrocytes increase after stimulation with LPS and IFNγ, so that these cells constitute a critical component in astrogliosis derived from an inflammatory stimulus (Mor et al., 2011). Moreover, miR-155 is an essential factor in microglia polarization into M1 profile, given that it blocks the transcription factor C/EBP-β, decreasing IL-10 production and consequently increasing NF-κB and iNOS activity (Vergadi et al., 2017). Thus, increased miR-155 transcription is related to a reduction in anti-inflammatory response, increasing inflammation (Gaudet et al., 2018). Despite the increase verified in miR-146a levels, miR-155 values were much higher, thus suppressing miR-146a anti-inflammatory role. These findings suggest that miR-155 levels increase in response to microglia activation, possibly playing a role in the fetal programming associated with the worse outcome verified in the EAE of ETS-exposed offspring. Corroborating our results, Wang et al. (2014) showed that exposure to ETS increased miR-155 expression in an atherosclerosis model. The literature has already described the manipulation of miR-155 levels in several neurodegenerative diseases, such as Amyotrophic Lateral Sclerosis, Alzheimer’s Disease, Parkinson’s Disease, and MS. In a study conducted by O’Connell et al. (2010), the authors reported that miR-155 inhibition or deletion in the murine model of EAE is neuroprotective, for the activation of helper and cytotoxic T cells decreases, as well as the production and release of cytokines.

A previous study has already described the association between stress during intrauterine life and increased risk of diseases in adulthood (Stevenson et al., 2020). Our study shows that *in utero* exposure to ETS stimulates aspects of neuroinflammatory response *in vitro*, such as microglia activation, cytokines and NO release, and elevated apoptosis. This scenario may increase the expression of pro-inflammatory miR-155 in microglia and astrocytes. Moreover, the increase in a fetal programming modulator may also be involved in the more severe outcome observed for ETS-exposed offspring during the EAE protocol.

## Data Availability

The raw data supporting the conclusions of this article will be made available by the authors, without undue reservation.
