# Supplementary material for: In Utero Exposure to Environmental Tobacco Smoke Increases Neuroinflammation in Offspring
Source: Front Toxicol. 2022 Jan 17;3:802542. doi: 10.3389/ftox.2021.802542 (PMC8915864; doi:10.3389/ftox.2021.802542)
Supplement: Supplementary file 1 [file DataSheet1.PDF]

Supplementary Table 1: Descriptive Statistical Analysis from experiments described on figures 2, 3 and 5.

	Challenge X exposure Interaction	Exposure effect	Challenge effect
Astrocytes (n°)	$F_{(1, 20)} = 4.94$; $p = 0.0380$	$F_{(1, 20)} = 49.78$; $p < 0.0001$	$F_{(1, 20)} = 16.70$; $p = 0.0006$
Microglia (n°)	$F_{(1, 20)} = 0.04$; $p = 0.8366$	$F_{(1, 20)} = 23.33$; $p = 0.0001$	$F_{(1, 20)} = 2.44$; $p = 0.1338$
MFI CD80	$F_{(1, 20)} = 2.32$; $p = 0.1437$	$F_{(1, 20)} = 0.92$; $p = 0.3484$	$F_{(1, 20)} = 159.60$; $p < 0.0001$
MFI CD86	$F_{(1, 20)} = 3.58$; $p = 0.0732$	$F_{(1, 20)} = 25.78$; $p < 0.0001$	$F_{(1, 20)} = 167.10$; $p < 0.0001$
IL-6 – qPCR	$F_{(1, 8)} = 71.46$; $p < 0.0001$	$F_{(1, 8)} = 62.02$; $p < 0.0001$	$F_{(1, 8)} = 822.10$; $p < 0.0001$
Inos – qPCR	$F_{(1, 8)} = 87.26$; $p < 0.0001$	$F_{(1, 8)} = 87.26$; $p < 0.0001$	$F_{(1, 8)} = 578.20$; $p < 0.0001$
Tlr4 – qPCR	$F_{(1, 8)} = 2.83$; $p = 0.1310$	$F_{(1, 8)} = 5.783$; $p = 0.0429$	$F_{(1, 8)} = 80.25$; $p < 0.0001$
Ikkα – qPCR	$F_{(1, 8)} = 4.41$; $p = 0.0689$	$F_{(1, 8)} = 3.01$; $p = 0.12$	$F_{(1, 8)} = 25.06$; $p = 0.0010$
IL-6 (pg/mL)	$F_{(1, 20)} = 11.19$; $p = 0.0032$	$F_{(1, 20)} = 11.19$; $p = 0.0032$	$F_{(1, 20)} = 15446$; $p < 0.0001$
IL-10 (pg/mL)	$F_{(1, 20)} = 1.22$; $p = 0.282$	$F_{(1, 20)} = 45.85$; $p < 0.0001$	$F_{(1, 20)} = 151.9$; $p < 0.0001$
IL-12 - (pg/mL)	$F_{(1, 20)} = 7.14$; $p = 0.0146$	$F_{(1, 20)} = 230.10$; $p < 0.0001$	$F_{(1, 20)} = 1.84$; $p = 0.19$
TNFα - (pg/mL)	$F_{(1, 20)} = 5.30$; $p = 0.0322$	$F_{(1, 20)} = 4.15$; $p = 0.0551$	$F_{(1, 20)} = 3756$; $p < 0.0001$
miRNA-155	$F_{(1, 8)} = 0.58$; $p = 0.4691$	$F_{(1, 8)} = 69.01$; $p < 0.0001$	$F_{(1, 8)} = 0.0007$; $p < 0.0001$
miRNA-146	$F_{(1, 8)} = 3.22$; $p = 0.1105$	$F_{(1, 8)} = 20.90$; $p = 0.0018$	$F_{(1, 8)} = 0.11$; $p = 0.751$
miRNA-223	$F_{(1, 8)} = 5.16$; $p = 0.0530$	$F_{(1, 8)} = 0.87$; $p = 0.379$	$F_{(1, 8)} = 12.75$; $p = 0.0073$

Supplementary Table 2: Descriptive Statistical Analysis from experiments described on figure 4.

	Minocycline X exposure X challenge interaction	Minocycline X exposure interaction	Minocycline X challenge interaction	Exposure effect	Challenge effect	Minocycline effect
Apoptosis (%)	$F_{1, 32)} = 3.66$; $p = 0.649$	$F_{(1,32)} = 28.27$; $p < 0.0001$	$F_{(1,32)} = 33.49$; $p < 0.0001$	$F_{(1,32)} = 5.49$; $p < 0.05$	$F_{(1,32)} = 31.04$; $p < 0.0001$	$F_{(1,32)} = 116.40$; $p < 0.0001$
Live cells (%)	$F_{(1, 32)} = 0.02$; $p = 0.89$	$F_{(1,32)} = 29.23$; $p < 0.0001$	$F_{(1,32)} = 27.72$; $p < 0.0001$	$F_{(1,32)} = 8.45$; $p < 0.01$	$F_{(1,32)} = 31.32$; $p < 0.0001$	$F_{(1,32)} = 116.10$; $p < 0.0001$
CCL2 (pg/mL)	$F_{(1, 32)} = 3.79$; $p = 0.060$	$F_{(1, 32)} = 3.96$; $p = 0.055$	$F_{(1,32)} = 77.00$; $p < 0.0001$	$F_{(1, 32)} = 1.16$; $p = 0.289$	$F_{(1,32)} = 237.00$; $p < 0.0001$	$F_{(1,32)} = 75.30$; $p < 0.0001$
TNFα (pg/mL)	$F_{(1, 32)} = 1.12$; $p = 0.297$	$F_{(1, 32)} = 1.53$; $p = 0.225$	$F_{(1,32)} = 43.54$; $p < 0.0001$	$F_{(1, 32)} = 0.0003$; $p = 0.97$	$F_{(1,32)} = 104.30$; $p < 0.0001$	$F_{(1,32)} = 40.10$; $p < 0.0001$
NO (µM)	$F_{(1, 32)} = 1.82$; $p = 0.187$	$F_{(1,32)} = 5.69$; $p < 0.05$	$F_{(1, 32)} = 1.05$; $p = 0.312$	$F_{(1,32)} = 7.74$; $p < 0.01$	$F_{(1,32)} = 6.80$; $p < 0.05$	$F_{(1,32)} = 19.70$; $p < 0.001$
